# A common copy-number variant within SIRPB1 correlates with human Out-of-Africa migration after genetic drift correction

**DOI:** 10.1371/journal.pone.0193614

**Published:** 2018-03-08

**Authors:** Jose Luis Royo, Joan Valls, Rafael D. Acemel, Carlos Gómez-Marin, Mariona Pascual-Pons, Arantxa Lupiañez, Jose Luis Gomez-Skarmeta, Joan Fibla

**Affiliations:** 1 Department of Basic Medical Sciences, IRBlleida, University of Lleida, Lleida, Spain; 2 Department of Surgery, Biochemistry and Immunology, University of Malaga, Malaga, Spain; 3 Biostatistics & Epidemiology Unit, IRBlleida, University of Lleida, Lleida, Spain; 4 Centro Andaluz de Biologia del Desarrollo, CSIC-Junta de Andalucia, Sevilla, Spain; Universitat Pompeu Fabra, SPAIN

## Abstract

Previous reports have proposed that personality may have played a role on human Out-Of-Africa migration, pinpointing some genetic variants that were positively selected in the migrating populations. In this work, we discuss the role of a common copy-number variant within the *SIRPB1* gene, recently associated with impulsive behavior, in the human Out-Of-Africa migration. With the analysis of the variant distribution across forty-two different populations, we found that the SIRPB1 haplotype containing duplicated allele significantly correlated with human migratory distance, being one of the few examples of positively selected *loci* found across the human world colonization. Circular Chromosome Conformation Capture (4C-seq) experiments from the *SIRPB1* promoter revealed important 3D modifications in the *locus* depending on the presence or absence of the duplication variant. In addition, a 3’ enhancer showed neural activity in transgenic models, suggesting that the presence of the CNV may compromise the expression of *SIRPB1* in the central nervous system, paving the way to construct a molecular explanation of the *SIRPB1* variants role in human migration.

## Introduction

In South East Africa, about 100,000 years ago (y.a.) the early Homo sapiens began its expansion [1,2]. According to mitochondrial DNA data, they followed the northeast coastline of Africa and continued their migration to the Middle East and next to Southern Asia and reached Australia. About 40,000 y.a., humans moved from Middle East north-west into Europe. Almost at the same time, modern humans crossed the Bering strait between Asia and North America and started a north-to-south expansion. The initial spread of humanity across the Earth was driven primarily by food and climate [3]. However, some authors have proposed that these movements may had not been merely due to environmental circumstances and probably some innate personality variables played a role on the final decisions. From a neurological point of view, there are evidences supporting that the prefrontal cortex is both associated to the planning and voluntary control of behavior [4–6]. To date, one of the best characterized personality-associated gene is Dopamine Receptor D_4_. *DRD4* functional variants have been found to be associated to novelty seeking, the personality trait associated with an exploratory activity [7]. Previous studies observed that populations who migrated farther in the past 30,000 to 1,000 y.a. had a higher frequency of these *DRD4* alleles and also showed that this differences can be also found between nomadic and sedentary populations [8]. Later results confirmed this hypothesis across different populations worldwide [9].

Recently, signal regulatory protein beta-1 (*SIRPB1*) was identified as a novel personality associated *locus* [10]. *SIRPB1* maps 20p13 and spans 64 Kb found expressed in the myeloid cells including microglia. Within *SIRPB1* intron 1, Laplana et al. identified a copy number variant of 30 kb that was associated to impulsive behavior [10]. Subjects with the ancestral allele were more impulsive than those with the duplicated allele. We hypothesized that if this CNV was contributing to personality scores within human population, these funcional alleles of *SIRPB1* may have played a role on human migration. To adress this question we took advantage of the genomic data from the *SIRPB1* locus of 42 populations available both from ALFRED [11] and the 1,000 genome project [12] to worldwide trace the presence of the CNV Duplication allele. This analysis revealed that *SIRPB1* CNV Duplication allele underwent a positive selection during the migratory events. In order to shed some light to the molecular mechanisms responsible for the behavioral changes, we performed comparative circular chromatin conformation capture experiments (4C-seq)[13] and demonstrated that the presence of the Duplication allele alters the local architecture of the chromatin modifying enhancer-promoter interactions.

## Materials and methods

### Population data

Genetic drift distances were based on the analysis of 501 randomly selected microsatellites [14]. Migratory distances from the 42 populations were calculated according to previously reported methods [9] using the geographical longitude and latitude coordinates from S1 Table and the migratory routes previously reported [1]. A total of 1,532 SNPs from the 2,536 participants of the 1,000 genome project (ftp://ftp.1000genomes.ebi.ac.uk//vol1/ftp/technical/reference/phase2_reference_assembly_sequence/hs37d5.fa.gz) were used in the first stage (hg19 coordinates chr20:1558613–1625882). Next, for the narrow SIRPB1 locus analysis was performed using rs2263664, rs2253698, rs2746603, rs6074896, rs2209313, rs1535882, rs11696842, rs6105421 and rs4814391 allele frequencies obtained from ALFRED database (http://alfred.med.yale.edu/alfred/). Selection of 10,000 random SNPs was used to calculate false discovery rate (FDR). Linkage dissequilibrium bloks differences where determined according to Hapmap release-3 data and visualized using Haploview [15]. Migratory distances were calculated using google earth software as previously described^9^ following the migration routes reported [16,17].

### SIRPB1 genotyping

Linkage between SIRPB1 CNV Duplication allele and rs2209313 was performed genotyping both polymorphisms in 555 subjects from six different geografical origins: South East Africa (Mozambique; n = 31), North Western Africa (Mali; n = 9, others; n = 17), Guinea Gulf Coast (Nigeria; n = 14, Ghana; n = 16; others; n = 5), East Asia (Taiwan; n = 24), North Eastern Eurone (Finland, n = 58), and South Central Europe (Austria; n = 65, and Spain; n = 331). These DNAs were available from laboratory collection reference C.0007431. SIRPB1 CNV genotype was determined according to a previously reported assay [18]. Genotyping of SIRPB1 rs2209313 marker was performed by Taqman assay C1911298. The study protocol conforms to the ethical guidelines of the Declarationof Helsinki and was approved by the Ethics Committee for Human Research of the University of Lleida and University Hospital Arnau de Vilanova. Written informed consent for enrolment in the study and for data management was obtained from all subjects in accordance with Spanish law.

### OOA migration statistical analysis

Generalized least square (gls) regression models were used to assess the association of the studied SNPs, using the arcsine transformation applied on the square root of the allelic proportions, with the migratory distance as the dependent variable. Models were estimated maximizing the log-likelihood instead of the restricted log-likelihood, assuming a Gaussian distribution. Correction for genetic drift was considered introducing a correlation matrix in the gls models, with no specific structure. Thus, a pseudo-correlation matrix, resulting from applying the transformation (max(D^2^)-D^2^)/max(D^2^) to the matrix containing the genetic distances (D^2^) between the 42 populations (S1 Fig), as kindly provided by Dr. Tishkoff was specifically considered in the models for genetic drift adjustment. Akaike’s Information Criterion was then used to evaluate the best model with a subset of main effects but also to determine the best models with any combination of SNPs interacting. To correct the obtained p-values for multiple testing we used the data from the set of randomly selected SNPs to compute the inflation in the type I error. Thus, we multiplied each p-value by an inflation factor defined as the ratio between the proportion of models found significant (p-value<0.05) in the testing set divided by the current singificance level, α = 0.05. To perform this correction, 10,000 SNPs or SNPs combinations were fitted to estimate the inflation FDR-correction factor. In addition, to evaluate the value of the association of *SIRPB1* SNPs on the migratory distance with relation to that previously reported for *DRD4* SNPs, we performed a sub-analysis considering the 13 populations where both SIRPB1 and DRD4 data was available. Different gls models containing both biomarkers were fitted also considering genetic drift adjustment.

### SIRP cluster region analysis

Whole Genome sequencing data from four different subjects [HGDP01029 (San), HGDP00521 (French), HGDP00542 (Papuan), HGDP00778 (Han) and HGDP00927 (Yoruba)] were obtained from UCSC genome browser (https://genome.ucsc.edu/Neandertal/). Evolutionary analysis of the SIRP cluster was conducted using MEGA6 [19]. The evolutionary history was inferred by using the Maximum Likelihood method based on the Tamura-Nei model [20]. Initial tree(s) for the heuristic search were obtained automatically by applying Neighbor-Join and BioNJ algorithms to a matrix of pairwise distances estimated using the Maximum Composite Likelihood approach, and then selecting the topology with superior log likelihood value.

### 4C-seq protocol and data analysis

Periferal blood mononuclear cells were obtained from six different healthy donnors (two for each of the genotypes studied), purified using Ficoll and rapidly fixed using paraformaldehyde (2% final concentration) for 10’ at room temperature. Fixation was then quenched with glycine (0.15M final concentration).The rest of the 4C-seq assays were performed as previously reported^13,21^. Isolated cells were lysed (lysis buffer: 10 mM Tris-HCl pH 8, 10 mM NaCl, 0.3% Igepal CA-630 (Sigma-Aldrich, I8896) and 1× protease inhibitor cocktail (Complete, Roche, 11697498001)), and the DNA was digested with DpnII (New England BioLabs, R0543M) and Csp6I (Fermentas, Thermo Scientific, FD0214) as primary and secondary enzymes, respectively. T4 DNA ligase (Promega, M1804) was used for both ligation steps. Second ligation was purified by dyalisis using Amicon Ultra-15 Centrifugal Filters (10,000 NMWL, Merck Millipore, UFC901024). Specific primers were designed around the putative transcriptional start site of the *SIRPB1* gene. Illumina adaptors were included in the primer sequences, and eight PCRs were performed with the Expand Long Template PCR System (Roche, 11759060001) and pooled. These libraries were purified with Agencourt AMPure XP magnetic beads (Beckman, A63880), their concentrations were measured using the Qubit dsDNA HS Assay Kit (Thermo Scientific, Q32851) and they were sent for single-end deep sequencing. 4C-seq data were processed as previously described [21]. Briefly, raw sequencing data were demultiplexed and aligned using the human reference genome (hg19). Reads located in fragments flanked by two restriction sites of the same enzyme, in fragments smaller than 40 bp or within a window of 10 kb around the viewpoint were filtered out. Mapped reads were then converted to reads per first enzyme fragment ends and smoothened using a 30-fragment mean running window algorithm. 4C-seq data were normalized by the total weight of reads within +-2Mb around the viewpoint. CTCF orientation track was obtained by merging the CTCF peaks of several publicly available CTCF ChIP-seq experiments from the ENCODE project (S2 Table) and looking for the CTCF motifs inside those peaks using JASPAR (http://jaspar.genereg.net/).

### Zebrafish enhancer assays

Zebrafish (*Danio rerio*) colonies have been maintained at the CABD Animal Facility according to previously stated procedures (http://zfin.org), in accordance with National and European regulations (S1 File). CABD animal facility is registered as animal research center with the number SE/4/U. Veterinary welfare supervision and daily water check-ups are conducted (dissolved oxygen, conductivity, pH, ammonia, nitrites, nitrates, alkalinity and hardness–Kh and Gh-, among other parameters) to ensure the animals good health status. Temperature, humidity and light intensity control in the room are strictly monitorized to guarantee animal welfare. The experimental zebrafish procedures have been performed following the protocols approved by the Ethical Committee for Animal Research from Junta de Andalucia. Two candidate regions, ENH1 and ENH2, from the 3’ end of *SIRPB1* were amplified (ENH1F: 5’-CTCTGGGGCTTTCTCTCCTT-3’ and ENH1R 5’-TTGACTCAGCCATTTTGCAG-3’) and ENH2F 5’-TCAGGATGAAACGTGGGATA-3’ ENH2R: 5’-GTGACCTCAGCCACCAGTCT-3’. Both regions were tested for enhancer activity as described in S1 File. Briefly, amplicons where cloned into pCR8/GW/TOPO vector (Invitrogen, Pasadena, USA) and recombined into the Zebrafish Enhancer Detection (ZED) shuttle vector which is based on the Tol2 transposase [22]. ZED vector contains the minimal GATA promoter upstream the enhanced green fluorescent protein (eGFP). eGFP expression is active whenever an enhancer element is present. ZED-vector also contains the cardiac actin promoter driving the expression of the red fluorescent protein (RFP), which serves as a positive control for transgenesis in F0 and F1 embryos [23].

### SIRPB1 allele-specific chromatin conformation capture

PBMCs of two heterozygous donnors were prepared as for 4C analysis with some modifications. Upon PFA fixation, samples were digested with HindIII (New England BioLabs, R0543M) and further ligated. Next, PCRs were performed using a common primer mapping SIRPB1 promoter (anchor 5’- CAATTGAGCTCTTCCTACCATGT-3’) and a sensor primer mapping the 3’ enhancer fragment (5’-TTCTCTTCCAGCATCCCATC-3’). PCR products had 1.8Kb and contanined rs2200313 whose C/T alleles were in linkage disequilibrium with the Dup/Anc alleles, respectively. To quantify the percentage of contacts between the enhancer and the promoter we determined the C/T ratios using Taqman assay C1911298 over the generated amplicons.

## Results

### Capturing *SIRPB1* CNV Duplication allele in different populations

**Fig 1:**
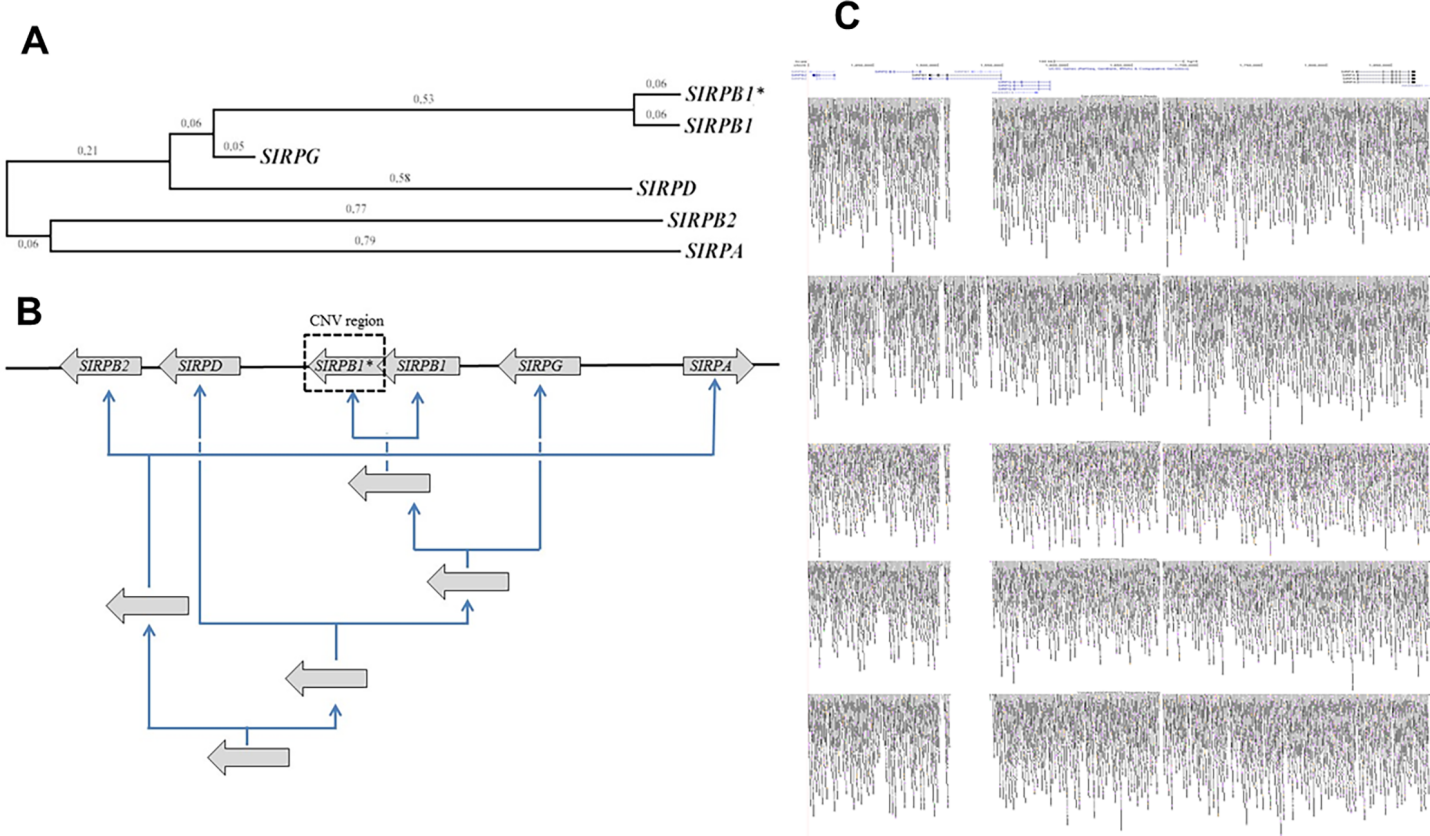
*Sirp* cluster analysis. (A) Molecular Phylogenetic analysis by Maximum Likelihood method. The evolutionary history was inferred by using the Maximum Likelihood method based on the Tamura-Nei model [20] Molecular Biology and Evolution 10:512–526]. The tree with the highest log likelihood (-95064.5787) is shown. Initial tree(s) for the heuristic search were obtained automatically by applying Neighbor-Join and BioNJ algorithms to a matrix of pairwise distances estimated using the Maximum Composite Likelihood (MCL) approach, and then selecting the topology with superior log likelihood value. The tree is drawn to scale, with branch lengths measured in the number of substitutions per site. The analysis involved 6 nucleotide sequences. All positions containing gaps and missing data were eliminated. There were a total of 14317 positions in the final dataset. Evolutionary analyses were conducted in MEGA6 [19]. (B) Diagram illustrating the above results takinq into account the current disposition of the cluster. (C) Genome-wide sequencing of four different subjects from (up-down order) HGDP01029 (San), HGDP00521 (French), HGDP00542 (Papuan), HGDP00778 (Han) and HGDP00927 (Yoruba). Data reflects a common deletion spanning 37Kb of *SIRPB1* intron 1.

**Fig 2:**
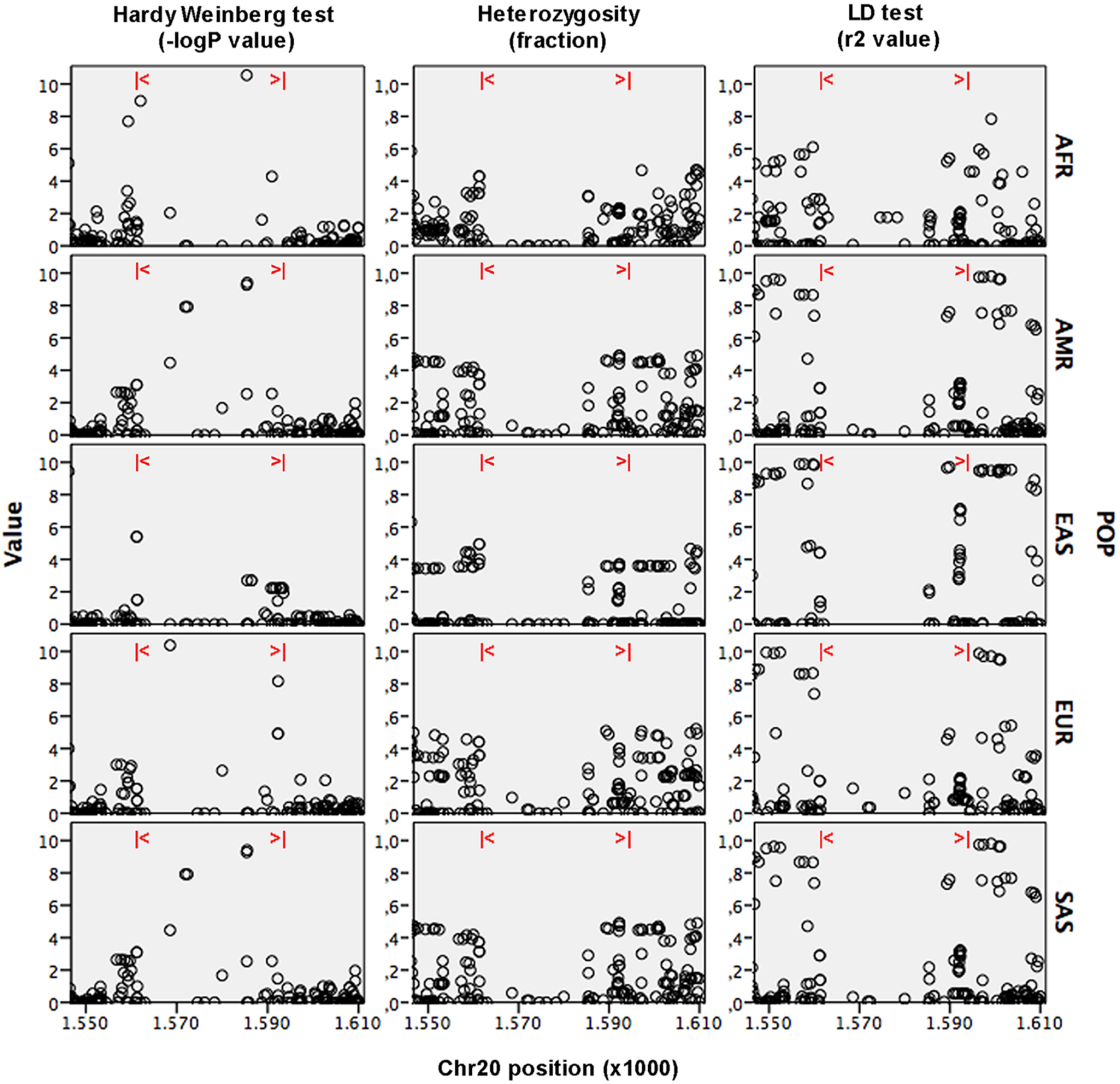
Local analisis of the SNPs covering the critical region. Left panel reflects the Hardy Weinberg equilibrium of the SIRPB1 region according to the 1.000 genome project data available for Africa (AFR), America (AMR), Europe (EUR), east and south Asia (EAS and SAS, respectively). Middle panel reflects the heterozygosity rates for the SNPs under analysis. Right panel illustrates the linkage disequilibrium analisis for each population, between the different SNPs and the *SIRPB1* CNV. The label (|<>|) shows the limits of the CNV.

**Fig 3:**
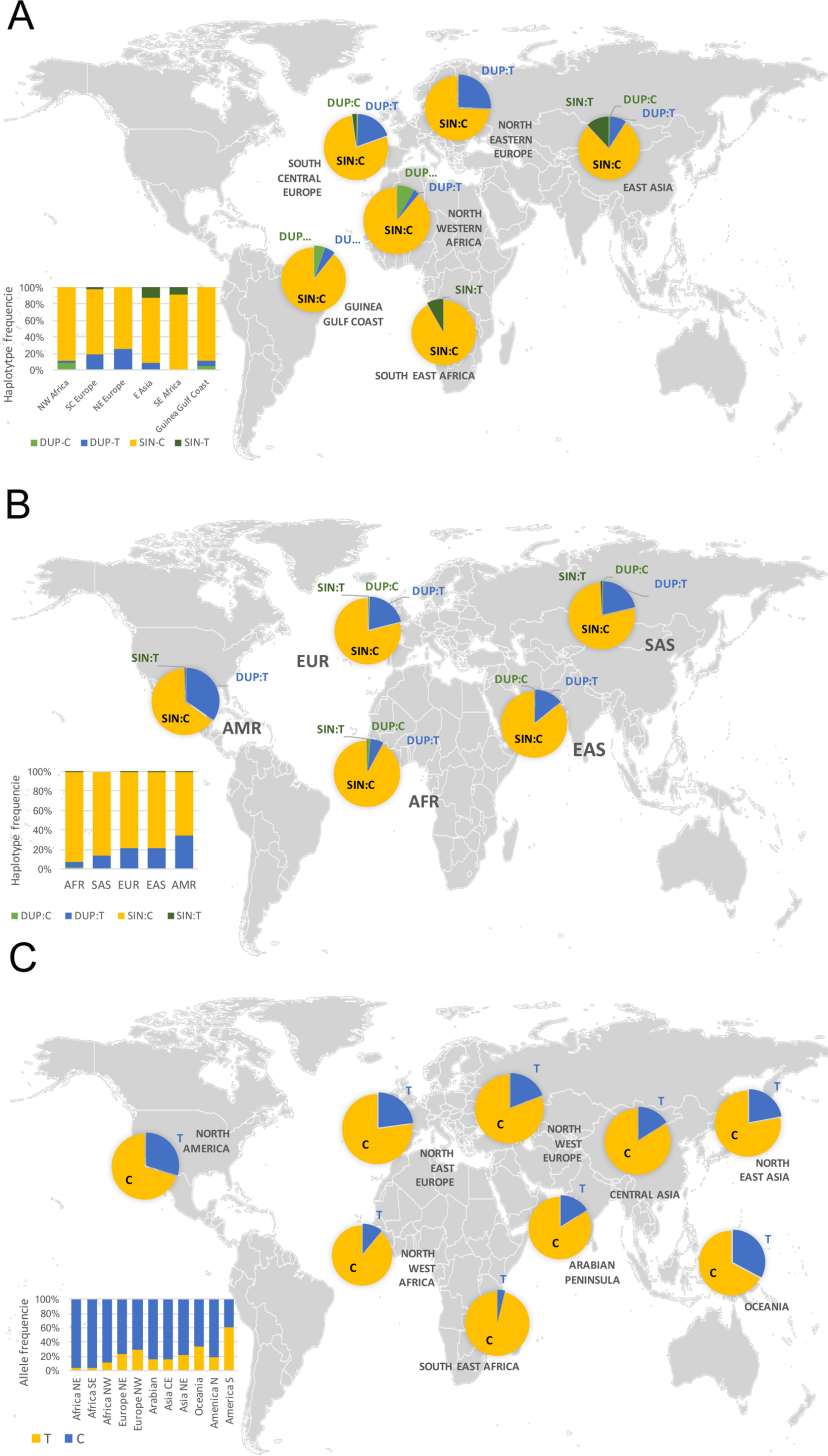
Worldwide distribution of the *SIRPB1* haplotypes. (A) Haplotype frequencies of rs2209313 and CNV obtained from different populations obtained in the laboratory. (B) Haplotype frequencies obtained in this study using the information available from the 1,000 genome project. (C) Worldwide distribution of rs2209313 frequency. Maps were obtained from the *maps* package from R version 3.2.0 available at https://CRAN.R-project.org.

In humans, the five genes of the SIRP family are grouped in a single cluster of 0.5 Mb on 20p13.1. Phylogenetic analysis using MEGA revealed that the insertion allele (Dup) of the CNV comes from a recent duplication of *SIRPB1* (Fig 1A and 1B) that can be directly visualized using UCSC genome browser in HGDP00521, one of the currently available fully sequenced donnors (Fig 1C). We investigated if this duplication occurred as a unique ancestral event and was therefore constrained to a single haplotype. We explored the region analyzing 1,532 SNPs and structural variants with a minimal allele frequency (MAF) >10% from 2,536 donnors of the 1,000 genome project. We observed a clear loss of heterozigosity within the CNV region, that generated significant deviations from the Hardy-Weinberg equilibrium (Fig 2). This effect was observed in every population analyzed and constrained to the region where the CNV was idenfied. Our LD analysis revealed that for European descents (EUR), Amerindians (AMR), East and South Asia (EAS, SAS) populations, the *SIRPB* CNV Duplication allele could be captured using different SNP combinations inlcluding rs1535882, rs4814391, rs6074896 and specially rs2209313 that was able to capture the CNV among the different populations (Fig 2). When CNV-rs2209313 haplotypes frequencies were generated, it could be observed that in South East Africa, the CNV Ancestral allele was mainly captured by rs2209313 allele C (hereafter, Sin-C) and was the most abundat haplotype with a frequency of 92%, while the Dup-T (CNV duplicated allele or Dup and rs2209313 allele T) haplotype frequency was 6.4% (Fig 3). The Dup-C haplotype showed a frequency of 1.5%, however while me move to northern populations, this haplotype is almost undetectable, as occurs to Sin-T. In order to validate these results we decided to genotype both rs2209313 and the CNV in six independent populations from two South and Western European cohorts (Spain and Austria), one series from East Asia (Taiwan) and two Africa series of including South East (Mozambique and Ruanda) and North Wertern (Mali, Guinea, Burkina, Senegal, etc). Our results mostly correlated with the ones obtained from the 1,000 genome project. rs2209313 allele-T captured the Duplication allele and *vice versa*. Some discrepancies were observed probably due to differences in the sample sizes. For instance, according to our data, in East Asia the Dup-T haplotype can be found with a frequency of 8%, in contrast to the 21% referred by the 1,000 genome project. Beyond these discrepancies, we noticed that the further we moved from South Central Africa, the more prevalent was the Dup-T haplotype. In order to address wether this could be due to simple genetic drift or may be the result of a positive selection we decided to conduct a systematic and quantitative approach.

### Genetic association of *SIRPB1* allele with human migratory distance after genetic drift correction

To confirm that the higher prevalence of the Duplication allele among the individuals that decided to migrate was not due to non-selective evolutionary forces, we modeled the distances covered during the Out of Africa (OOA) migration by the 42 different populations as a function of the proportion of the *SIRPB1* CNV genotype, taking into account the genetic drift. First of all we quantified the distances covered between 42 populations, according to the routes that were followed during the OOA migration. Next, based on the LD information obtained for the *SIRPB1* locus for all the previously analyzed populations, we selected the SNPs flanking the CNV that were able to capture the Duplication event according to the five populations available from the 1,000 genome data and evaluated their frequencies across the 42 populations (Table 1). Next, in order to take into consideration the genetic drift, we used Tishkof’s matrix of evolutionary distances between populations based on the neutral distribution of microsatellites to correct against genetic drift [14].

**Table 1:** Allele frequencies among the studied populations and their respectives OOA migration distances.

POPULATION	Distance (Km)	rs2263664	rs2253698	rs2746603	rs6074896	rs2209313	rs1535882	rs11696842	rs6105421	rs4814391
San	0	0,75	0,75	1,00	0,83	1,00	0,83	0,83	1,00	1,00
Mbuti	2716	0,97	0,80	1,00	0,87	1,00	0,83	0,83	0,97	1,00
Bantu speakers	2920	0,93	0,73	1,00	0,65	0,95	0,83	0,95	0,93	0,87
Biaka	2934	0,92	0,87	1,00	0,79	1,00	1,00	1,00	0,97	1,00
Yoruba	3900	0,94	0,58	1,00	0,67	0,94	0,90	0,92	0,94	0,98
Mandenka	5574	0,90	0,83	1,00	0,50	0,89	0,85	0,88	0,85	1,00
Bedouin	6400	0,94	0,47	0,99	0,66	0,90	0,67	0,89	0,98	0,78
Mozabite	6470	0,88	0,67	0,91	0,63	0,92	0,68	0,92	0,97	0,90
Palestinian	6482	0,92	0,58	0,98	0,68	0,95	0,78	0,95	0,97	0,84
Druze	6696	0,89	0,79	0,99	0,68	0,72	0,68	0,72	0,98	0,96
Adygei	7840	0,82	0,65	0,97	0,62	0,88	0,82	0,88	1,00	0,94
Italians	8708	0,88	0,67	1,00	0,72	0,88	0,71	0,88	1,00	0,81
Sardinian	8886	0,91	0,55	0,96	0,80	0,74	0,59	0,75	1,00	0,84
Balochi	9099	0,89	0,58	0,97	0,70	0,85	0,74	0,85	1,00	0,89
Brahui	9285	0,86	0,52	0,98	0,52	0,88	0,86	0,88	0,98	0,98
French	9411	0,79	0,64	0,97	0,79	0,72	0,53	0,72	1,00	0,81
Sindhi	9783	0,84	0,58	0,93	0,72	0,80	0,68	0,80	1,00	0,88
Basque	9878	0,77	0,54	0,85	0,71	0,75	0,58	0,75	1,00	0,83
Kalash	9967	0,90	0,90	1,00	0,88	0,86	0,84	0,86	1,00	0,98
Orcadian	10513	0,66	0,66	0,97	0,78	0,63	0,44	0,63	1,00	0,81
Uyghur	10945	0,85	0,85	1,00	0,80	0,90	0,90	0,90	1,00	1,00
Tu	12147	0,70	1,00	1,00	1,00	0,75	0,75	0,75	1,00	1,00
Russians	12164	0,76	0,54	0,88	0,62	0,70	0,60	0,70	1,00	0,90
Mongolian	12551	0,55	1,00	1,00	1,00	0,56	0,60	0,60	1,00	1,00
Dai	12873	0,70	0,95	1,00	1,00	0,72	0,75	0,75	1,00	1,00
Lahu	12895	0,65	0,95	1,00	0,95	0,65	0,65	0,65	1,00	1,00
Yi	13022	0,70	0,95	1,00	0,90	0,65	0,65	0,65	1,00	1,00
Miao	13126	0,70	1,00	1,00	1,00	0,70	0,70	0,70	1,00	1,00
Daur	13380	0,78	0,89	0,89	0,89	0,72	0,72	0,72	1,00	1,00
Han	13400	0,89	1,00	1,00	1,00	0,89	0,89	0,89	1,00	1,00
Tujia	13654	0,85	1,00	1,00	1,00	0,85	0,85	0,85	1,00	1,00
Cambodians, Khmer	13803	0,91	0,95	1,00	0,91	0,77	0,77	0,77	1,00	1,00
Oroqen	13803	0,65	0,95	1,00	0,95	0,70	0,70	0,70	1,00	1,00
Hezhe	14141	0,67	1,00	1,00	1,00	0,81	0,83	0,83	1,00	1,00
Naxi	14147	0,83	0,89	1,00	0,89	0,83	0,83	0,83	1,00	1,00
She	14203	0,85	0,90	1,00	0,90	0,85	0,85	0,85	1,00	1,00
Japanese	15379	0,77	1,00	1,00	0,98	0,79	0,79	0,79	1,00	1,00
Papuan New Guinean	18323	1,00	0,79	1,00	1,00	1,00	0,82	1,00	1,00	1,00
Melanesian, Nasioi	19515	0,92	0,84	1,00	1,00	0,95	0,84	0,95	1,00	1,00
Pima, Mexico	21788	0,44	0,98	1,00	0,98	0,46	0,46	0,46	1,00	1,00
Maya, Yucatan	23603	0,48	0,90	0,98	0,94	0,52	0,50	0,52	1,00	0,98
Karitiana	28012	0,35	1,00	1,00	1,00	0,42	0,42	0,42	1,00	1,00
Surui	28336	0,33	1,00	1,00	1,00	0,38	0,38	0,38	1,00	1,00

The best model, the one with the lowest Akaike information criterion (AIC), included the main effects of 4 associated SNPs and their second order of interactions. As prevoulsy reported by Matthews et al, we used 10,000 randomly selected markers to correct for type I error [9]. After this correction, two SNPs remained significant: rs2209313 and rs6074896 (Table 2). Since population data do not provide haplotypic data, to further analyze the relationship of *SIRPB1* SNPs and migratory distance we assessed all possible models containing second and third order statistical interactions. As indicated in Table 2, the joint effect of rs2209313 with rs6074896 showed the lowest AIC, and was signiticantly associated to the migratory distances after multiple testing correction. Therefore, taking into account our previus LD studies demonstrating the association between rs2209313 allele-T and the CNV Duplication allele, we can conclude that it underwent a positive selection during OOA migration.

**Table 2:** Correlation between *SIRPB1* variants and human OOA distance.

	No genetic drift adjustment			With genetic drift adjustment		
	Beta (SE)	p-value	AIC	Beta (SE)	p-value	AIC
Individual effects (univariate models)						
rs1535882	18287.506 (4405.783)	0.0001	905.43	5060.443 (2646.23)	0.06	871.01
rs2209313	-18605.59 (3085.946)	<0.00001	893.81	-5318.516 (2529.557)	0.04	870.28
rs4814391	12849.663 (4673.758)	0.009	913.08	2552.277 (3180.968)	0.43	874.01
rs6074896	15899.344 (2632.256)	<0.00001	894.06	3235.918 (2401.931)	0.18	872.82
Significant main effects and 2-order interactions						
rs1535882	-21435.94 (40001.35)	0.6		-32915.59 (19620.29)	0.10	
rs2209313	1478.87 (33686.19)	0.97		-43507.25 (16800.97)	0.01	
rs4814391	81001.99 (25789.23)	0.003				
rs6074896	-119015.95 (27717.34)	0.0001		-50546.42 (23420.52)	0.04	
rs1535882*rs2209313	-34734.55 (13516.49)	0.01				
rs1535882*rs6074896	55484.95 (23664.94)	0.02		30591.29 (17164.91)	0.08	
rs2209313*rs4814391	-69475.19 (21604.17)	0.002				
rs2209313*rs6074896	87817.42 (17184.35)	<0.00001	717.11	33963.34 (14180.97)	0.02	869.63

### *SIRPB1* CNV alters local chromatin topology and correlates with differential gene expression

**Fig 4:**
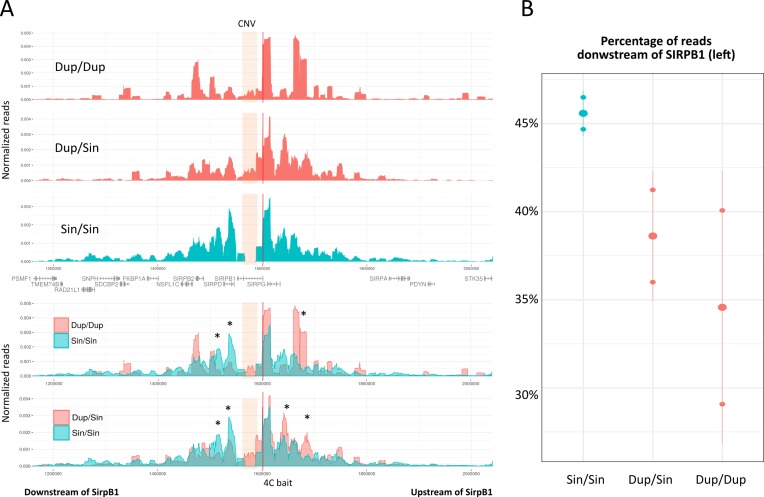
Cromatin conformation study of the *SIRPB1* locus. (A) Above the gene track, 4C-seq data from the *SIRPB1* promoter in the three genotypes studied. Below we present the overlays of the 4C-seqs presented above. 4C-seq viewpoint are represented by a red vertical line and the CNV region is shaded. Asterisks in the overlay highlight regions of consistently enriched contacts when comparing homozygous Sin profiles with the other two genotypes. (B) Quantification of the 4C-seq contact distribution at each side of *SIRPB1* promoter in the three genotypes studied.

After finding the correlation between the duplicated allele and the migratory behavior we explored possible molecular mechanisms associated to the presence of this duplication. Previous data showed that two CTCF binding sites with insulator activity were present within the CNV region and that their absence correlated with *SIRPB1* mRNA upregulation in peripheral blood cells [10]. It has been shown that CTCF plays a major role in maintaining the architecture of the human genome [24] and that perturbations of CTCF sites can severely distort gene regulation depending on distal enhancers [25]. Moreover, the CTCF motifs found in the CTCF sites of the CNV and the *SIRPB1* promoter were oriented in a convergent manner suggesting that they were interacting each other (S2 Fig). Therefore, we hypothesized that the presence of the duplicated region might alter the contacts between *SIRPB1* promoter and potential 3’ enhancers. To investigate this potential role of the Duplication event over chromatin interactions between both sides of the CNV we performed 4C-seq experiments from the *SIRPB1* promoter in Peripheral Blood Mononuclear Cells (PBMCs) of two healthy donnors of each of the genotypes of interest: Dup/Dup, Dup/Sin, Sin/Sin. In agreement with the presence of insulating CTCF sites in the CNV, we observed a decay in the contacts downstream of *SIRPB1* promoter (crossing the CNV) in heterozygous and Dup homozygous individuals (38.6%and 34.6% respectively) in comparison with homozygous Sin individuals (45.6%, Spearman p-value = 0.038, Fig 4). According to this observation, we propose that the weaker contacts downstream of *SIRPB1* in Dup/Dup individuals may be difficulting regulatory interactions between the promoters of genes including *SIRPB1* and enhancers located across the CNV. However, it remains to be elucidated which are these regulatory elements and which tissues are affected.

**Fig 5:**
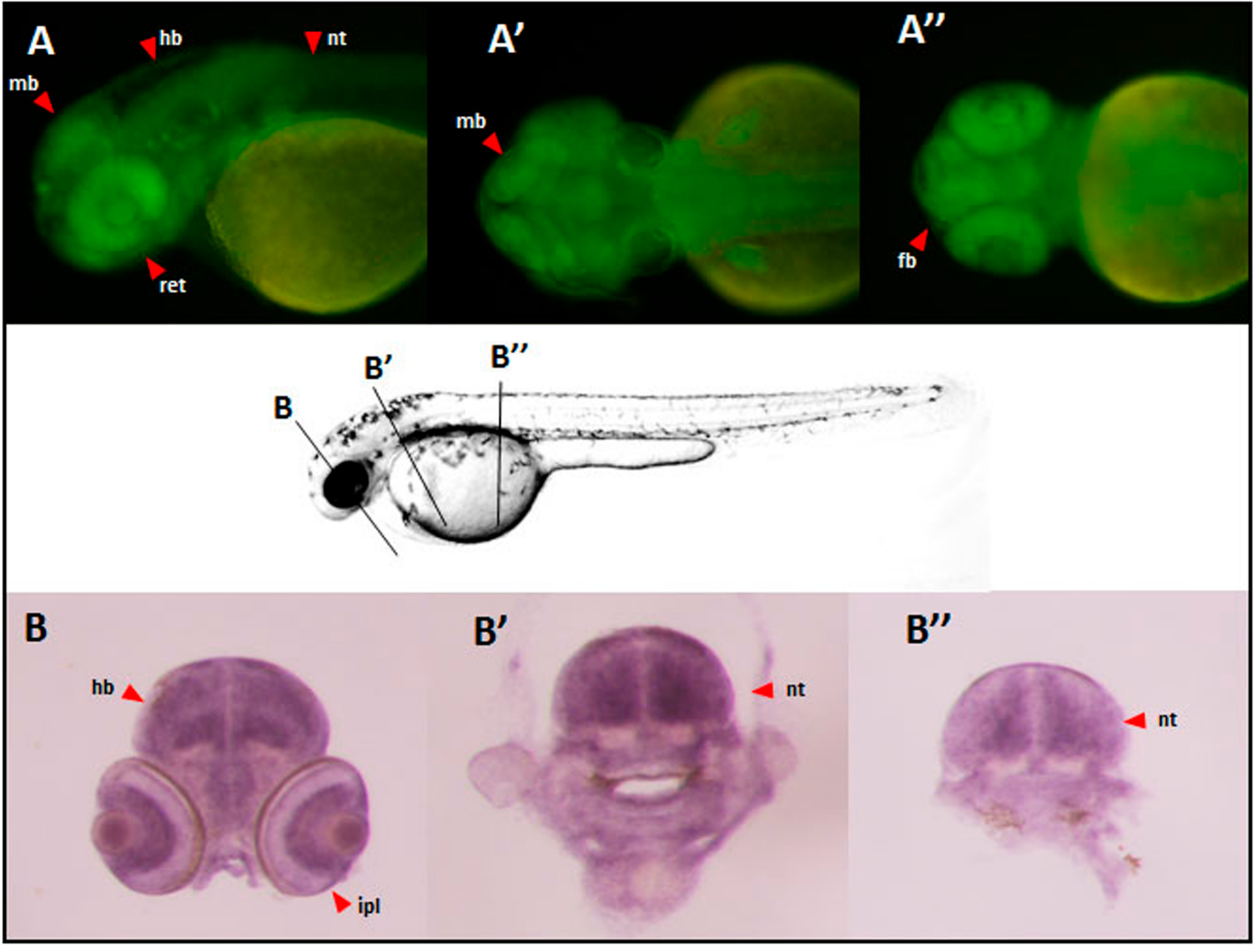
Enhancer activity of the 3’ side of *SIRPB1*. Enhancer anctivity of human ENH1 in transgenic zebrafish. Panel A shows a lateral view of a 36hpf transgenic zebrafish were the ENH1 enhancer activity is shown. GFP is expressed in the midbrain (mb), hindbrain (hB) and the neural tube (nt). A’ and A” are dorsal and ventral pictures, respectively. Whole mount *in situ* hybridization analysis illustrated in panels B, B’ and B”, correcponding to different sections of the transgenic Zebrafish. Expression in the inner plexiform layer (ipl) and the galglion cell layer is shown in panel B. Neural tube expression is evidenced in sections B’ and B”.

**Fig 6:**
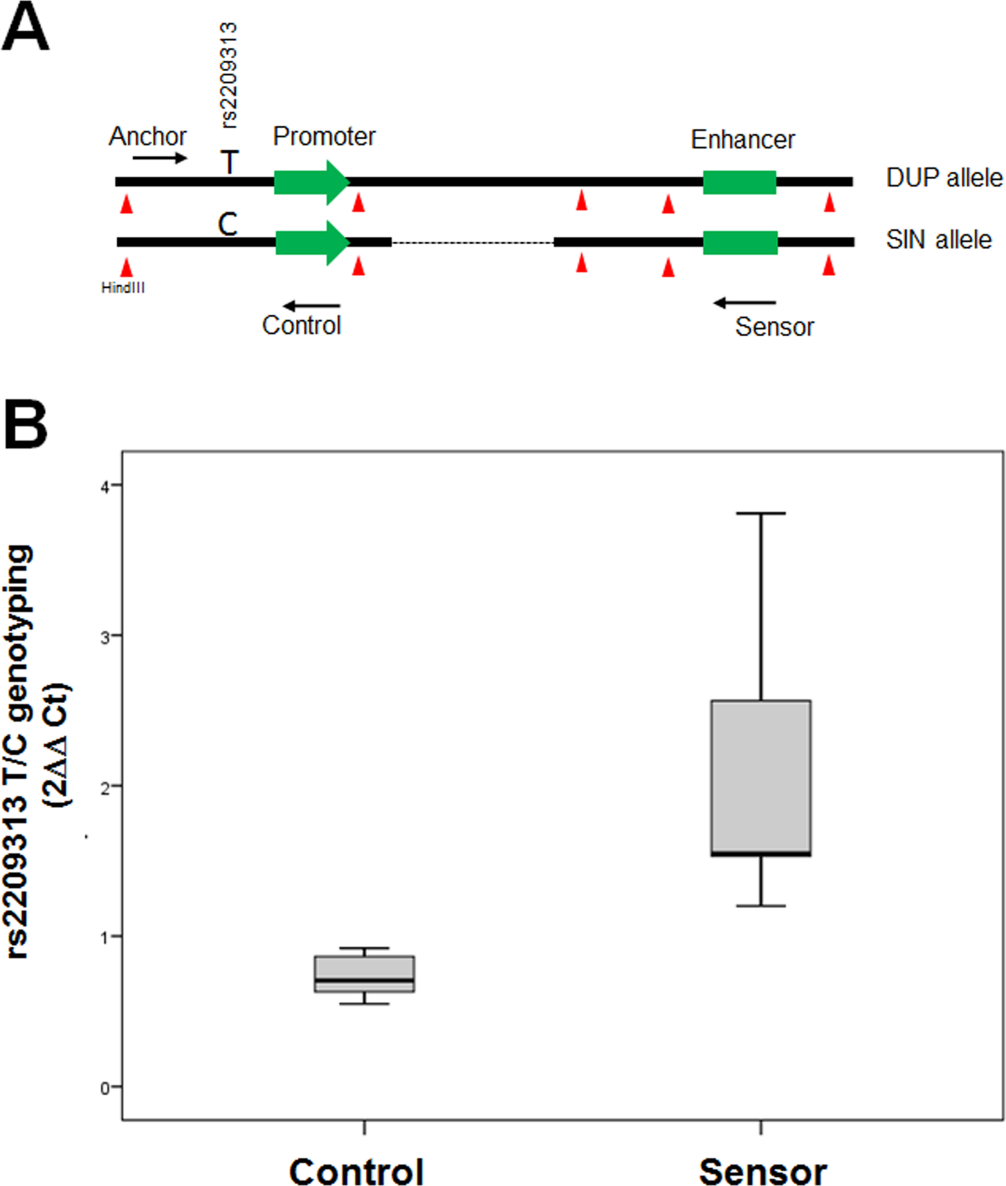
Allele-specific 3C analysis of *SIRPB1*. Panel A illustrates the design of the allele-specific 3C. The anchor primer is intended to amplify the rs2203313 before reaching the HindIII site. As a control, a reverse primer is designed to amplify the SNP before the amplicon reaches the HinddIII site to avoid any potential genotyping bias. Panel B show the results obtained from the genotyping using either the control or the sensor primer. Data were obtained after obtaining the Cts determined for C and T alleles.

Analyzing the epigenetic information available at UCSC genome browser, we observed two candidate regions from the 3’ site of *SIRPB1* that show H3K27 acetilation. These human regions were amplified and their enhancer capacity was tested with transgenic assays in Zebrafish using the ZED vector as previously reported. When stable F1 were analyzed we identified that ENH1 (hg19 chr20:1547859–1548906) exhibited a clear enhancer activity with the capacity to drive eGFP to different areas of the central nervous system (Fig 5). Beyond this neural activity, we decided to test wether this enhancer was really contacting *SIRPB1* promoter and to which extend these contacts were distorsioned by the presence of the duplication allele of the CNV. To this end we performed allele-specific 3C assays taking advantage of the position of rs2209313 near the *SIRPB1* proximal promoter and its LD with the CNV. Using this approach we were able to determine in a heterozygous donnor the relative percentage of contacts between the 3’ enhancer and the promoter depending on the allele. As illustrated in Fig 6, under normal conditions the ΔCt measured as Allele C Ct—Allele-T _Ct_ was close to zero (2^ΔCt^ = 1). However, when analyzing the relative abundance of contacts using a sensor primer mapping the 3’ end of *SIRPB1*, the C/T ratio showed a bias. The enhancer from the chromatide harboring the duplicated allele, herecaptured by the rs2209313-T allele showed a lesser efficient contact (higer Ct) than the one with the ancestral allele (identified as the rs2209313-C) (n = 6, p-value = 1.55x10^-4^, U-Mann Whitney test).

## Discussion

Personality traits are influential in individual decision making. From an evolutionary perspective, exploratory behavior could be seen as a mean to expand a community frontier. Some authors proposed that within societies with non-explorative behavior, people do not explore new food variants adequately and therefore suffer from malnutrition [26]. However, migration requires a psychic cost related to incertitude, this is, the potential risk of finding significant differences between the known environment and a future destination. Personality traits alter the individuals’ own perception of these psychic costs. Consequently, individuals having certain traits tend to migrate more. Different studies have investigated this phenomenon in contemporary populations. Fouarge et al investigated the relationship between the big personality traits and individuals’ migration intentions and found that extraversion positively associates with the intention to migrate [27]. This underlies that psychic costs associated with migration differ across individuals as their personality traits affect how much they perceive these costs under an environmental stress. Thus, personality traits are an integral part of cost minimization process if we assume that individuals try to maximize their expected utility from migration, and according to the rationale followed in this work, this phsycological networks might have been present during the modern human expansion.

Genetic data on the worldwide distribution of *DRD4* VNTR allele frequencies among different populations have been compiled from a number of genetic studies, drawing mainly from studies reporting allele frequencies from healthy individuals. Wang et al. found the 4-repeat to be the ancestral allele, and showed that two functionally diffetent alleles with 2 and 7 repeats emerged through positive selection associated to novelty seeking phenotype [28]. Thus was initially described analyzing *DRD4* VNTR frequencies comprised 1,327 individuals from 36 populations that did not undergo any recent genetic mixing with other populations [29]. From a functional perspective, Wang et al. proposed a model of selection acting on the *DRD4* VNTR with known biochemical and physiological differences between receptor variants [28]. Their argumentation is that different responses of *DRD4* alleles to dopamine levels may account for OOA migratory behavior, resulting in different human personality types prevailing under different environmental conditions. It has been suggested by Jensen et al.[30] and Wang et al.[28] that resource-depleted, time-critical, or rapidly changing environments might select for individuals with “response-ready” adaptations, whereas resource-rich, time-optimal, or little-changing environments might select against such adaptations. Maybe subjects with a strong inclination towards “response-ready” adaptations, such as novelty-seeking behavior, drove the wave of migration out of Africa [31].

The rationale underling our analysis is that personality-associated *loci* may underwent positive selection at the time of human OOA migration. We recently found that a common Duplication within *SIRPB1* introl 1 that was associated to a better impulsivity control. This is a multifactorial psychological construct that involves a tendency to act without reflection, or consideration of the consequences. Impulsive decisions are typically poorly conceived, risky, or inappropriate to the situation. This tipically results in undesirable consequences. Thus, we wondered if the Duplicated allele, associated to an increased impulsivity control, may have undergone a positive selection while human expansion. First, using both 1,000 genome project data and our own samples we determined that rs2209313 allele T is in LD with the *SIRPB1* CNV Duplication allele. The latter paved the way to a wider analysis, takin advantage of the greater amount of data on SNP frequencies. Thus, we observed an increasing frequence of the *SIRPB1* Dup-rs2209313-T haplotype as we move away from East Africa. Using the allele frequencies from 42 polulations populations and according to our LD data we observed that the *SIRPB1* Duplication allele underwent a non-random positive selection of across human populations after multiple corrections. In an attempt to gain further insight on this phenomenon we performed a molecular characterization of the CNV. Previous results highlighted the presence of two insulators within the critical region. Now our results show that the 3’ side of the CNV contains a neural enhancer that contacts *SIRPB1* promoter that may partially explain the expression of SIRPB1 in the brain. From the allele-specific 3C assays we know that the presence of the duplication affects cromatin structure altering the contacts between the 3’ proximal enhancer and *SIRPB1* promoter. However, *SIRPB1* expresssion seems not to be just controlled by this enhancer, since 4C experiments evidence multiple contacts points between *SIRPB1* promoter and the vicinity. Beyond this, 4C analysis suggest that the presence of the Duplication affects not only the proximal region, but the entire surrounding chromatin and therefore affecting the transcriptional regulation of *SIRPB1*. To our knowledge, this is the first CNV described to undergo a positive selection during human migration. However, additional independent studies are needed to confirm this hypothesis and determine whether this observation can be attributed to *SIRPB1* or another affected neighbor gene.

## Supporting information

S1 Fig Dendrogram showing the genetic distance between the populations under study. (JPG)

S2 Fig CTCF binding motif orientation is depicted in between the 4C-seq profiles of the allele without the duplication (top, Sin/Sin) and the heterozigous allele (bottom, Dup/Sin). Red arrowheads represent motifs in the plus (+) strand while blue arrowheads are motifs present in the minus (-) strand. The only motif found within the CNV is located in the + strand facing the CTCF site near the SIRPB1 promoter located in the—strand. Contacts between these two CTCF sites might be difficulting others with more distal elements as it is represented with the arrows scheme.
(TIF)

S1 Table Geographical coordinates from the studied populations. (DOCX)

S2 Table CTCF ChIP-seq experiments from the ENCODE project used. (XLSX)

S1 File Supporting methods. Animal care and transgenesis.
(DOCX)

## References

[1] Henn B.M., Cavalli-Sforza L.L., & Feldman M.W. The great human expansion. *Proc Nat Acad Sci USA*. 109,7758–7764 (2012). 10.1073/pnas.1212380109 PMC3497766 23077256

[2] White T.D., Asfaw B., DeGusta D., Gilbert H., Richards G.D., Suwa G., et al Pleistocene Homo sapiens from Middle Awash, Ethiopia. *Nature*. 423, 742–747 (2003). doi: 10.1038/nature01669 12802332 10.1038/nature01669

[3] Cavalli-Sforza L.L. & Feldman M.W. The application of molecular genetic approaches to the study of human evolution. *Nat Genet*. 33, Suppl:266–275(2003). 12610536 10.1038/ng1113

[4] Wilson G.D., Kumari V., Gray J.A., & Corr P.J. The role of neuroticism in startle reactions to fearful and disgusting stimuli. *Pers Individ Dif*. 29, 1077–1082 (2000).

[5] Kumari V., Ffytche D.H., Das M., Wilson G.D., Goswami S., & Sharma T. Neuroticism and brain responses to anticipatory fear. *Behav Neurosci*. 121, 643–652 (2007). doi: 10.1037/0735-7044.121.4.643 17663590 10.1037/0735-7044.121.4.643

[6] Drabant E.M., Kuo J.R., Ramel W., Blechert J., Edge M.D., Cooper J.R., et al Experiential, autonomic and neural responses during threat anticipation vary as a function of threat intensity and neuroticism. *Neuroimage*. 55, 401–410 (2011). doi: 10.1016/j.neuroimage.2010.11.040 21093595 10.1016/j.neuroimage.2010.11.040 PMC3031673

[7] Munafò M.R., Yalcin B., Willis-Owen S.A., & Flint J. Association of the dopamine D4 receptor (DRD4) gene and approach-related personality traits: meta-analysis and new data. *Biol Psyc*. 63, 197–206 (2008). 10.1016/j.biopsych.2007.04.006 17574217

[8] Chen C.S., Burton M., Greenberger E., & Dmitrieva J. Population migration and the variation of dopamine D4 receptor (DRD4) allele frequencies around the globe. *Evol Hum Behav*. 20, 309–324 (1999).

[9] Matthews L.J., & Butler P.M. Novelty-seeking DRD4 polymorphisms are associated with human migration distance out of Africa after controlling for neutral population gene structure. *Am J Phys Anthropol*. 145, 382–389 (2011). doi: 10.1002/ajpa.21507 21469077 10.1002/ajpa.21507

[10] Laplana M., Royo J.L., García L.F., Aluja A., Gomez-Skarmeta J.L., & Fibla J. 2014 SIRPB1 copy-number polymorphism as candidate quantitative trait locus for impulsive-disinhibited personality. Genes Brain Behav. 13(7):653–62. doi: 10.1111/gbb.12154 25039969 10.1111/gbb.12154

[11] Cheung K.H., Osier M.V., Kidd J.R., Pakstis A.J., Miller P.L., & Kidd K.K. ALFRED: an allele frequency database for diverse populations and DNA polymorphisms. *Nucleic Acids Res*. 28, 361–363 (2000). 10592274 10.1093/nar/28.1.361 PMC102486

[12] Auton A., Brooks L.D., Durbin R.M., Garrison E.P., Kang H.M., Korbel J.O., et al 1000 Genomes Project Consortium. A global reference for human genetic variation. *Nature*. 526, 68–74 (2015). doi: 10.1038/nature15393 26432245 10.1038/nature15393 PMC4750478

[13] Splinter E., de Wit E., van de Werken H.J., Klous P., & de Laat W. Determining long-range chromatin interactions for selected genomic sites using 4C-seq technology: from fixation to computation. *Methods*. 58, 221–30 (2012). doi: 10.1016/j.ymeth.2012.04.009 22609568 10.1016/j.ymeth.2012.04.009

[14] Tishkoff S.A., Reed F.A., Friedlaender F.R., Ehret C., Ranciaro A., Froment A., et al The genetic structure and history of Africans and African Americans. *Science*. 324, 1035–1044 (2009). doi: 10.1126/science.1172257 19407144 10.1126/science.1172257 PMC2947357

[15] Barrett J.C., Fry B., Maller J., & Daly M.J. Haploview: analysis and visualization of LD and haplotype maps. *Bioinformatics*. 21, 263–265 (2005). doi: 10.1093/bioinformatics/bth457 15297300 10.1093/bioinformatics/bth457

[16] Mellars P. Going East: new genetic and archaeological perspectives on the modern human colonization of Eurasia. *Science*. 313, 796–800 (2006). doi: 10.1126/science.1128402 16902130 10.1126/science.1128402

[17] Liu H., Prugnolle F., Manica A., & Balloux F. A geographically explicit genetic model of worldwide human-settlement history. *Am J Hum Genet*. 79, 230–237 (2006). doi: 10.1086/505436 16826514 10.1086/505436 PMC1559480

[18] Royo J.L., Pascual-Pons M., Lupiañez A., Sanchez-López I., & Fibla J. Genotyping of common SIRPB1 copy number variant using Paralogue Ratio Test coupled to MALDI-MS quantification. *Mol Cell Probes*. 29, 517–521 (2015). doi: 10.1016/j.mcp.2015.07.009 26239731 10.1016/j.mcp.2015.07.009

[19] Tamura K., Stecher G., Peterson D., Filipski A., & Kumar S. MEGA6: Molecular Evolutionary Genetics Analysis version 6.0. *Mol Biol Evol*. 30, 2725–2729 (2013). doi: 10.1093/molbev/mst197 24132122 10.1093/molbev/mst197 PMC3840312

[20] Tamura K., & Nei M. Estimation of the number of nucleotide substitutions in the control region of mitochondrial DNA in humans and chimpanzees. *Mol Biol Evol*. 10, 512–526 (1993). doi: 10.1093/oxfordjournals.molbev.a040023 8336541 10.1093/oxfordjournals.molbev.a040023

[21] Noordermeer D., Leleu M., Splinter E., Rougemont J., De Laat W., & Duboule D. The dynamic architecture of Hox gene clusters. *Science*. 334, 222–225 (2011). doi: 10.1126/science.1207194 21998387 10.1126/science.1207194

[22] Kawakami K., & Noda T. Transposition of the Tol2 element, an Ac-like element from the Japanese medaka fish Oryzias latipes, in mouse embryonic stem cells. *Genetics*. 166, 895–899 (2004). 15020474 10.1093/genetics/166.2.895 PMC1470731

[23] Bessa J., Tena J.J., de la Calle-Mustienes E., Fernandez-Minan A., Naranjo S., Fernández A., et al Zebrafish enhancer detection (ZED) vector: A new tool to facilitate transgenesis and the functional analysis of cis-regulatory regions in zebrafish. *Dev Dyn*. 238, 2409–2417 (2009). doi: 10.1002/dvdy.22051 19653328 10.1002/dvdy.22051

[24] Rao S.S., Huntley M.H., Durand N.C., Stamenova E.K., Bochkov I.D., Robinson J.T., et al A 3D map of the human genome at kilobase resolution reveals principles of chromatin looping. *Cell*. 159, 1665–1680 (2014). doi: 10.1016/j.cell.2014.11.021 25497547 10.1016/j.cell.2014.11.021 PMC5635824

[25] Lupiáñez DG, Kraft K, Heinrich V, Krawitz P, Brancati F, Klopocki E, et al Disruptions of topological chromatin domains cause pathogenic rewiring of gene-enhancer interactions. *Cell*. 161, 1012–1025 (2015). doi: 10.1016/j.cell.2015.04.004 25959774 10.1016/j.cell.2015.04.004 PMC4791538

[26] Williams J., & Taylor E. The Evolution of Hyperactivity, Impulsivity and Cognitive Diversity. *J R Soc Interface*. 3, 399–413 (2006). doi: 10.1098/rsif.2005.0102 16849269 10.1098/rsif.2005.0102 PMC1578754

[27] Fouarge D., Özer M., & Seegers P. Personality traits and migration intention: Who bears the cost of migration? Maastricht University, mimeo (2016).

[28] Wang E., Ding Y.C., Flodman P., Kidd J.R., Kidd K.K., Grady D.L., et al The Genetic Architecture of Selection at the Human Dopamine Receptor D4 (DRD4) Gene Locus. *Am J Hum Genet*. 74, 931–944 (2004). doi: 10.1086/420854 15077199 10.1086/420854 PMC1181986

[29] Chang F.M., Kidd J.R., Livak K.J., Pakstis A.J., & Kidd K.K. The World-Wide Distribution of Allele Frequencies at the Human Dopamine D4 Receptor Locus. *Human Genetics*. 98, 91–101 (1996). 8682515 10.1007/s004390050166

[30] Jensen P., Mrazek D., Knapp P.K., Steinberg L., Pfeffer C., Schowalter J., et al Evolution and Revolution in Child Psychiatry: ADHD as a Disorder of Adaptation. *J Am Acad Child Adolesc Psychiatry*. 36, 1672–1679 (1997). doi: 10.1097/00004583-199712000-00015 9401328 10.1097/00004583-199712000-00015

[31] Ding Y.C., Chi H.C., Grady D.L., Morishima A., Kidd J.R., Kidd K.K., et al Evidence of Positive Selection Acting at the Human Dopamine Receptor D4 Gene Locus. *Proc Nat Acad Sci USA* 99, 309–314 (2002). doi: 10.1073/pnas.012464099 11756666 10.1073/pnas.012464099 PMC117557

