# Supplementary figures and images for: A common copy-number variant within SIRPB1 correlates with human Out-of-Africa migration after genetic drift correction
Source: PLoS One. 2018 Mar 8;13(3):e0193614. doi: 10.1371/journal.pone.0193614 (PMC5843225; doi:10.1371/journal.pone.0193614)

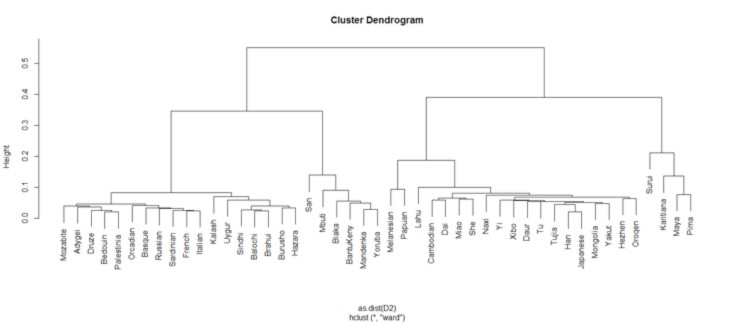

Supplement: S1 Fig — (JPG) [file pone.0193614.s001.jpg]

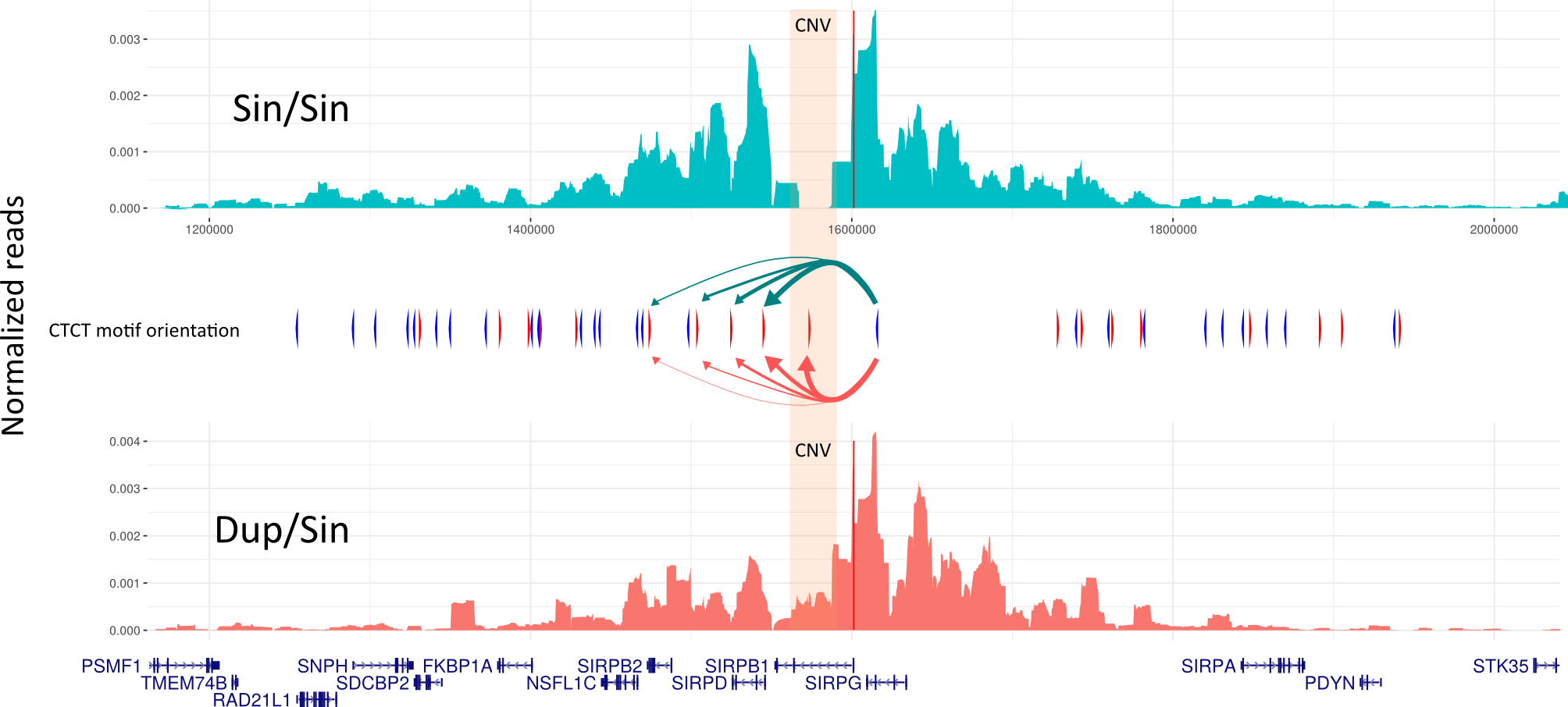

Supplement: S2 Fig — CTCF binding motif orientation is depicted in between the 4C-seq profiles of the allele without the duplication (top, Sin/Sin) and the heterozigous allele (bottom, Dup/Sin). Red arrowheads represent motifs in the plus (+) strand while blue arrowheads are motifs present in the minus (-) strand. The only motif found within the CNV is located in the + strand facing the CTCF site near the SIRPB1 promoter located in the—strand. Contacts between these two CTCF sites might be difficulting others with more distal elements as it is represented with the arrows scheme. (TIF) [file pone.0193614.s002.tif]
