# Supplementary material for: A common copy-number variant within SIRPB1 correlates with human Out-of-Africa migration after genetic drift correction
Source: PLoS One. 2018 Mar 8;13(3):e0193614. doi: 10.1371/journal.pone.0193614 (PMC5843225; doi:10.1371/journal.pone.0193614)
Supplement: S1 Table — (DOCX) [file pone.0193614.s003.docx]

**Supplementary Table 1. Geographical coordinates from the studied populations**

| **Population** | **Region** | **Longitude** | | **Latitude** | |
| --- | --- | --- | --- | --- | --- |
|  |  | **Max** | **min** | **max** | **min** |
| Bantu speakers | Africa | 18W | 51E | 37N | 35S |
| Biaka | Africa | 15E | 20E | 5N | 2N |
| Mandenka | Africa | 14W | 12W | 14,5N | 12,5N |
| Mbuti | Africa | 26E | 30E | 3N | 0N |
| Mozabite | Africa | 0 | 7E | 34N | 31N |
| San | Africa | 13E | 26E | 15S | 26S |
| Yoruba | Africa | 2E | 8E | 10N | 6N |
| Balochi | Asia | 57E | 68E | 31N | 25N |
| Bedouin | Asia | 34E | 36E | 32,25N | 29,25N |
| Brahui | Asia | 61E | 68E | 30N | 25N |
| Druze | Asia | 35E | 37E | 32,5N | 34N |
| Kalash | Asia | 71E | 75E | 37N | 33N |
| Mongolian | Asia | 88E | 122E | 53N | 38N |
| Palestinian | Asia | 34E | 36,5E | 33N | 30N |
| Sindhi | Asia | 63E | 75E | 31N | 20N |
| Cambodians, Khmer | EastAsia | 102,5E | 107,5E | 14,5N | 10,5N |
| Dai | EastAsia | 96,5E | 106E | 28N | 21N |
| Daur | EastAsia | 97E | 135E | 53,5N | 37,5N |
| Han | EastAsia | 100E | 120E | 40N | 22N |
| Hezhe | EastAsia | 121,5E | 135E | 53,5N | 43N |
| Japanese | EastAsia | 130E | 146E | 46N | 30N |
| Lahu | EastAsia | 97E | 106E | 29N | 21N |
| Miao | EastAsia | 96,5E | 114E | 34N | 21N |
| Naxi | EastAsia | 109,5E | 122,5E | 33N | 20N |
| She | EastAsia | 109,5E | 122,5E | 31N | 20N |
| Tu | EastAsia | 90E | 105E | 40N | 31,5N |
| Tujia | EastAsia | 108E | 116E | 34N | 25N |
| Uyghur | EastAsia | 72E | 97E | 50N | 35N |
| Yi | EastAsia | 97E | 112E | 35N | 21,5N |
| Adygei | Europe | 39E | 40,5E | 45N | 44N |
| Basque | Europe | 3,5W | 1W | 43,75N | 42,5N |
| French | Europe | 3E | 7,5E | 51N | 42,5N |
| Italians | Europe | 7E | 18,5E | 37,9N | 47N |
| Orcadian | Europe | 3,5W | 2,75W | 59,5N | 58,75N |
| Russians | Europe | 30E | 180E | 85N | 45N |
| Sardinian | Europe | 8E | 10E | 41,25N | 38,75N |
| Maya, Yucatan | NorthAmerica | 90W | 88W | 20N | 18N |
| Pima, Mexico | NorthAmerica | 109,5W | 108W | 30,5N | 28N |
| Melanesian, Nasioi | Oceania | 154E | 156E | 5S | 7S |
| Papuan New Guinean | Oceania | 130,5E | 154E | 0,5S | 11,5S |
| Karitiana | SouthAmerica | 65W | 63,5W | 9S | 9,5S |
| Surui | SouthAmerica | 62W | 60W | 9S | 11,5S |
