# Supplementary material for: A common copy-number variant within SIRPB1 correlates with human Out-of-Africa migration after genetic drift correction
Source: PLoS One. 2018 Mar 8;13(3):e0193614. doi: 10.1371/journal.pone.0193614 (PMC5843225; doi:10.1371/journal.pone.0193614)
Supplement: S1 File — Animal care and transgenesis. (DOCX) [file pone.0193614.s005.docx]

**Supplementary methods**

**Animal care**

Zebrafish (Danio rerio) were maintained and obtained from our breeding colony under standard conditions according to previously stated procedures (http://zfin.org). Embryos for Tol2 transgenesis were obtained from crosses of wild-type AB/Tuebingen (AB/TU) zebrafish. Potential transgenic founders were out-crossed to a TAP strain. Fertilized eggs were kept at 28°C in E3 medium with 0.003% 1-phenyl-2-thiourea to prevent pigmentation and were staged according to Kimmel et al (1995).

**Transgenic enhance testing**

PCR fragments where amplified using HiFi Taq polymerase (Roche, Manheim, Germany) using standard PCR procedures. Products where cloned into pCR8/GW/TOPO vector (Invitrogen, Pasadena, USA). Clones where recombined into the Zebrafish Enhancer Detection (ZED) shuttle transgenesis vector previously described [Bessa J, 2009]. Briefly, ZED-Vector contains two modules flanked by the Medaka (Oryza latipes) Tol2 transposase target sites, what enables an efficient transgenesis (Kawakami et al. 2004). The first module contains the minimal GATA promoter driving the expression of the enhanced green fluorescent protein (EGFP). All HCNRs were cloned upstream of this module using the Gateway system (Invitrogen, Pasadena, USA). Two strong insulators, which reduce the potential influence of the regulatory elements that may be present in the vicinity of the integration sites, flank this reporter cassette. The second module contains the cardiac actin promoter driving the expression of the red fluorescent protein (RFP), which serves as a positive control for transgenesis in F0 and F1 embryos (Bessa J, 2009).

A minimum of 300 embryos where injected with 3–5 nl of a solution containing 25 nM of each construct and 25 nM of Tol2 mRNA. Embryos where then incubated at 28°C as previously described. EGFP expression was evaluated 24, 48 and 72 hours post-fertilization (hpf). Whenever EGFP was observed, the HCNR tested was considered as a potential candidate and embryos were selected and raised to sexual maturity to be analyzed in F1. The efficiency of the integration of the ZED-HCNR construct in the injected embryos was determined by the expression of RFP in the somites and the heart. We only evaluated the enhancer potential of the HCNRs when RFP was broadly observed in the somites and the heart of the injected embryos, as an indication of efficient ZED-HCNR integration. For high-resolution pictures a F-View black/white digital camera coupled to a WD70 Nikon camera was used. Adobe Photoshop was used to adjust bright and contrast.

**Suppementary references.**

Kimmel CB, Ballard WW, Kimmel SR, Ullmann B, Schilling TF. Stages of embryonic development of the zebrafish. Dev Dyn. 1995;203:253–310

Kawakami K. Transgenesis and gene trap methods in zebrafish by using the Tol2 transposable element. Methods Cell Biol. 2004;77:201–222.
